# Successful Transplantation of Human Bone

**Published:** 1881-10

**Authors:** 


### SUCCESSFUL TRANSPLANTATION OF HUMAN BONE.

The *Glasgow Medical Journal* informs us that at the meeting of the Pathological and Clinical Society of that city, April 12th, 1881, Dr. William Macewen showed a patient on whom transplantation of human bone had been performed, whereby over two-thirds of the shaft of the right humerus had been restored. The grafts were taken from six wedges of bone removed from limbs of patients affected with antero-tibial curves, and were reduced to very small fragments previously to insertion. The patient was formerly shown to the Society after the first graft had been completed, when there was a restoration of the upper part of the shaft, to the extent of one inch in length. Now, the shaft was completely restored, and the right humerus only measured one half-inch shorter than the left.

